# Immunophenotypic Characteristics and Cytogenetic Analysis of Adolescent and Young Adult B-Cell Acute Lymphoblastic Leukemia: Correlations With Clinicopathological Parameters

**DOI:** 10.7759/cureus.68735

**Published:** 2024-09-05

**Authors:** Vineeta Yadav, Veeramani Raveendranath, Prasanth Ganesan, Rakhee Kar, Priyadharshini R, Prabhu Manivannan

**Affiliations:** 1 Anatomy, Jawaharlal Institute of Postgraduate Medical Education & Research, Puducherry, IND; 2 Oncology, Jawaharlal Institute of Postgraduate Medical Education & Research, Puducherry, IND; 3 Pathology, Jawaharlal Institute of Postgraduate Medical Education & Research, Puducherry, IND; 4 Pharmacology, Jawaharlal Institute of Postgraduate Medical Education & Research, Puducherry, IND

**Keywords:** adolescent and young adult (aya), b-all, cd marker, flow cytometery, fluorescence in situ hybridization (fish), mrd

## Abstract

Introduction

Acute lymphoblastic leukemia (ALL) has suboptimal survival rates for adolescents and young adults (AYA) as compared to children. Very limited studies have been conducted on AYA patients in India. This study aimed to identify the cytogenetic and immunophenotype characteristics of B-cell ALL (B-ALL) in AYA patients and determine its correlation with clinicopathological parameters in the Southern India region.

Method

The study was a prospective study conducted for three years, from June 2019 to May 2022, in India. Newly diagnosed 90 patients with AYA (13-40 years) ALL were recruited. A B-ALL diagnosis was made based on morphology with cytochemical stains and immunophenotype by flow cytometry (FCM). Cytogenetic analysis was also performed using karyotyping and fluorescent in situ hybridization to identify chromosomal aberrations. The cytogenetics results were correlated with immunophenotyping and clinicopathological characteristics. Variables were analyzed using the Mann-Whitney U test and Chi-square test using IBM SPSS Statistics for Windows, Version 20.0 (Released 2011; IBM Corp., Armonk, NY, USA).

Results

The mean age was 22.68 ± 8.06 years. It was observed that the most common structural chromosomal abnormality for AYA was t(9;22) in 14 (15%) cases, followed by 6q deletions in seven (8%) cases, t(1;19) in four (4%) cases, and t(12;17) and t(6;14) in two (2%) cases each. In addition, t(3;12), t(2;11), t(12;21), t(1;9), t(2;12), and t(X;10) were found in one (1%) case each. The most common numerical abnormality was hyperdiploidy (15; 17%), followed by hypodiploidy (10; 11%). Further, myeloid antigen expression of CD33 was the most common aberrantly expressed marker found in 20 (28%) cases, followed by CD15 in three cases (5%), CD13 in three (4%) cases, and CD11b in two (3%) cases. It was also observed that in Ph+ve cases, CD33 and CD13 were most commonly expressed in three (33%) and two (17%) cases, respectively. In contrast, in Ph-ve patients, their expressions were lesser at 17 (27%) and one (2%) cases, respectively. In addition, leukemia-associated immunophenotype pattern (LAIP) markers CD44 6 (86%) and CD123 5 (55%) were also found to be significantly associated with Ph+ve, whereas their values in the Ph-ve group were lesser at 25 (42%) and 9 (17%), respectively. Our data also showed that older age wassignificantly associated with Ph+ve with a median age of 30 years (p = 0.012). In comparison, the median age of Ph-ve was only 21 years.

Conclusion

Our study established that the incidence of cytogenetic abnormalities for AYA was consistent with previously reported data. This study reaffirms that Ph+ve cases have significant associations with MyAg (CD13), LAIP (CD123 and CD44), and older age for the South Indian population.

## Introduction

Acute lymphoblastic leukemia (ALL) is characterized by abnormal cell proliferation and immature blast cell aggregation in the bone marrow and peripheral blood [1]. The clinical profile of ALL presents an infiltration of leukemic blasts in extramedullary sites leading to lymphadenopathy, splenomegaly, and hepatomegaly with symptoms of anemia, thrombocytopenia, and leukopenia. Age, white blood cell count, blast cell counts at the end of induction, karyotype, and immunophenotype are the most essential parameters of prognostic significance in ALL [2].

Based on the cell lineage, 80% of ALL cases are B-cell ALL (B-ALL), and the remaining are T-ALL. ALL predominantly affects children and is bimodal in distribution, with the first peak at two to five years of age and the second peak at around 50 years [3]. The median age of diagnosis of ALL is 14 years, and around 60% of cases are identified at <20 years, 25% at 45 years, and 11% at around 65 years [4]. The incidence of ALL in adolescents and young adults (AYA) (defined by the National Comprehensive Cancer Network NCCN as 15-39 years) and adults is one-third of that in children [1,5,6]. The five-year survival rate in children is excellent at about 80-90%. In contrast, survival rates are suboptimal at around 20-40% in adults [3]. The prognosis is 62.2% in 15-19 years of age and 52.8% in 20-39 years of age [7,8]. The remarkably inferior outcome in AYA compared to their younger counterparts is reported by several studies in Western countries; however, such studies are scarce in India.

In this study, we have targeted the AYA age group in the South Indian population. AYA is identified as a unique population with exclusive characteristics and requirements. Different regions (countries) and organizations have different definitions of age limits for AYA. These differences affect access to care structures and treatment trials or protocols [8]. AYA is also associated with increased high-risk factors and decreased favorable prognostic elements. Issues such as challenges in recruiting this specific age group, lack of clinical trials, and inferior outcomes due to poor adherence to treatment protocol in constrained settings have also been highlighted in earlier studies [9-11]. Enigma about proper diagnosis and therapeutic implications can be the reason for poor prognosis and inferior outcomes in the AYA age group [8].

It is well acknowledged that cytogenetics is crucial in ALL patients’ diagnosis, prognosis, and risk stratification. Cytogenetic analysis identifies chromosomal rearrangements, which can be structural, numerical, or a combination. Structural rearrangements include translocation, inversion, deletion, and duplication, whereas numerical rearrangements comprise the gain or loss of chromosomes [1]. In ALL, chromosomal aberrations such as translocation (especially t(9;22)), inversion, deletions, and hypodiploidy are associated with an unfavorable prognosis [12]. These abnormal karyotypes are the initial events of leukemogenesis and also provide a base for studying the molecular mechanism of leukemic blast and uncontrolled cell proliferation [2,12,13]. Thus, in this study, we have used cytogenetics to find the incidence of chromosomal abnormalities in the South Indian population.

Along with cytogenetics, FCM-based immunophenotyping is a vital pillar of the WHO leukemia classification, substantially impacting diagnosis, lineage characterization, and subclassification of hematologic malignancies [14]. In addition to the diagnosis, it gives invaluable information about prognosis and helps decide appropriate treatment strategies [15]. The combined information of chromosomal abnormality and aberrant myeloid expression (obtained from immunophenotyping) provides great insight into the biology of leukemic blast and the disease. Further, the combination of peculiar immunophenotypes of leukemic cells and specific chromosomal rearrangements shows a distinctive outcome in ALL [16]. Understanding and awareness of the association between cytogenetics and immunophenotype provides a quick and cost-effective diagnosis and paves the way to plan treatment strategies, especially in a resource-constrained setting [14]. Minimal data is available for such association in AYA B-ALL cases in the South Indian population. Thus, this study aims to address this vital gap for the critical group AYA population (known for its suboptimal survival outcomes) by expressing the correlation between genetic rearrangements and aberrant myeloid expression in B-ALL. In addition, this study also reports the clinicopathological characteristics of the target AYA population.

## Materials and methods

Study design and setting

The study was a prospective investigation conducted at the Jawaharlal Institute of Postgraduate Medical Education & Research, Puducherry, India, over a three-year period from June 2019 to May 2022. All suspected ALL cases in the AYA age group (13-40 years) were recruited from the Department of Medical Oncology. The diagnosis of B-ALL was made by assessment of morphology along with immunophenotyping by FCM. The institutional review board approved the protocol (approval number JIP/IEC/2018/365). Signed informed consent was obtained from the patients who were ≥18 years old or from the parents or guardians of younger patients, with assent from the patients, as appropriate. T-ALL cases were excluded as part of the exclusion criteria for the study.

Sample collection

A 1-2 ml sample of bone marrow aspirate was collected, with one portion placed in an EDTA tube for flow cytometry (FCM) and another in a Sodium Heparin vacutainer for cytogenetic analysis. Smears were prepared directly or from the EDTA sample and stained with Giemsa for morphological evaluation (baseline parameters) and immunophenotyping via FCM. Cytochemical stains, including Sudan Black B and periodic acid-Schiff, were also performed.

Cytogenetics analysis

Karyotyping

Fresh bone marrow aspirate samples collected in a sodium heparin-anticoagulated vacutainer were used for cytogenetic studies as described below. The first step was to take the WBC count, after which the samples were immediately processed for a short time culture (24 hours). Following the standard harvesting and slide preparation protocol, G-banding was done using Giemsa stain.

Slides were scanned under a light microscope for metaphases. Based on chromosome morphology, good spreads, and banding quality, a minimum of 20 metaphases were captured from two or three slides of each sample. Analysis was done under Olympus-BX53 Microscope by using IKAROS software.

Structural and numerical abnormalities were interpreted per the standard International System for Human Cytogenetic Nomenclature.

Fluorescent In Situ Hybridization (FISH)

FISH analysis for the t(9;22), t(12;21), and t(4;11) translocations was performed on both interphase nuclei and metaphase chromosomes using the Meta Systems BCR/ABL1 plus dual color probe. Analyses were performed on BM cells prepared according to conventional cytogenetic techniques and placed on slides. The hybridization and denaturation process with fluorochrome-labeled FISH probes was performed with slight modifications according to the protocol provided by manufacturers. Fluorescence signals were evaluated using an Olympus BX53 fluorescence microscope in a 100X oil objective. A minimum of 100 cells/samples were counted and captured.

FCM

The stain-lyse-wash procedure was used for all samples. The sample was diluted with PBS if the WBC count was more than 50,000/ul. In all these cases, a minimum of 100,000 events were acquired in a 3-laser 10-color Navios flow cytometer (Beckman Coulter company). The analysis was performed using Kaluza software. The panel of CD markers used in FCM is shown in Table 1. In the first step, an acute leukemia orientation tube was performed to indicate the lineage of leukemia. B1, B2, and B3 tubes were further processed only if B-lineage was shown. The panel of antibodies, their fluorochrome, and the clone used are shown in Table 2.

**Table 1:** Panel of CD markers used in flow cytometry LAIP, leukemia-associated immunophenotype pattern

Myeloid markers	B lineage markers	T lineage markers	LAIP markers
CD13	CD10	sCD3	CD9
CD33	CD19	cCD3	CD44
CD15	CD20	CD5	CD58
CD11b	CD22	CD7	CD73
CD64	CD79a	CD8	CD81
CD14	-	-	CD86
CD117	-	-	CD123
cMPO	-	-	-

**Table 2:** Panel of CD markers used for B cell-ALL characterization X denotes the absence of an antibody in that panel. ALL, acute lymphoblastic leukemia; ALOT, acute leukemia orientation tube

Fluorochrome	FITC	PE	ECD	PC5.5	PC7	APC	APC 700	APC 750	PB	KO
ALOT tube	nTdT	cMPO	CD19	cCD79a	CD34	cCD3	CD45	CD7	sCD3	HLA-DR
B1 tube	CD81	X	CD123	CD19	CD34	CD10	CD20	CD38	CD9	CD45
B2 tube	CD58	CD73	CD19	CD86	CD34	CD10	CD20	CD38	CD44	CD45
B3 tube	CD13	CRLF2	CD19	CD33	CD34	CD11c	CD45	X	CD11b	CD15

Sometimes, due to the unavailability of some markers at the time of sample processing in a particular case, such markers were not performed. To avoid errors in results due to the nonavailability of a particular marker, such cases have been excluded while working out the percentages for each marker. This has been achieved by varying the denominator to the actual number of cases for each marker.

Antigen expression was reported per WHO style-tripartite consensus reporting (negative, weak, or strong) [17]. If more than 10% of the leukemic blast cells were positive for a particular antigen, they were assigned as positive for that marker. Positive expression was further qualified as strong if the expression was ≥50% and weak if it was in the 10-50% range. Expression of <10% was designated as negative.

Statistical analysis

Statistical analysis was performed using IBM SPSS Statistics for Windows, Version 20.0 (Released 2011; IBM Corp., Armonk, NY, USA). Continuous variables were analyzed using the Mann-Whitney test, and categorical variables were analyzed using the Chi-square test. A p-value of less than 0.05 was considered significant.

## Results

Cytogenetics

Cytogenetic studies were performed in 90 B-ALL cases. Based on the presence of t(9;22), cytogenetic groups were classified into Ph+ve and Ph-ve. Out of 90 cases, 14 (15%) were diagnosed as Ph+ve, and 76 (84%) were identified as Ph-ve. Normal karyotype was found in 46 (51%) cases. t(9;22) was the most common structural chromosomal abnormality reported for AYA in 14 (15%) cases, followed by 6q deletions in seven (8%) and t(1;19) in four (4%) cases. The most common numerical abnormality was hyperdiploidy (15; 17%), followed by hypodiploidy (10; 11%). The other structural and numerical chromosomal abnormalities observed are listed in Table 3.

**Table 3:** Identified chromosomal abnormalities

Abnormality	Total number of cases observed, n (%)	Common abnormalities
46,XX/46,XY (Normal karyotype)	46 (51%)	-
t(9;22) (q34;q11)	14 (15%)	46,XX,t(9;22) n=2, 46,XY,t(9;22) n=6, 55,XY,+X,+4,+5,+6,+8,+11,+14,+17,+21,+22,t(9;22) n=1, 46-47,XY,+4,+5,t(9;22) n=1, 45,XX,-7,t(9;22) n=2, 45,XX,del(6q),-7,t(9;22) n=1, 45,XY,del(6q),-7,t(9;22) n=1
46,XY t(12;21)	1 (1%)	-
t(6;14)	2 (2%)	46,XY,t(6;14) n=1, 46,XX,t(X;10),t(6;14) n=1
t(1;19)	4 (4%)	46,XX,t(1;19) n=3, 46-47,XX,+4,t(1;19) n=1
46,XX t(X;10), t(6;14)	1 (1%)	-
44-47 XY, +4,+5 del(6 ),t(2;12)	1 (1%)	-
46,XY t(2;11)	1 (1%)	-
46,XY t(1; 9)	1 (1%)	-
46,XY t(12;17) and 46,XX t(12;17)	2 (2%)	-
46,XY, del(6q), t(3;12)	1 (1%)	-
6q del	7 (8%)	46,XY,del(6) n=1, 46,XX,del(6) n=1, 46-47,XY,+4,del(6) n=1, 45,XX,del(6q),-7,t(9;22) n=1, 45,XY,del(6q),-7,t(9;22) n=1, 46,XY,del(6q),t(3;12) n=1, 44-47,XY,+4,+5,del(6),t(2;12) n=1
Hyperdiploidy	15 (17%)	46-47,XX,+4 n=3, 46-47,XX,+4,t(1;19) n=1, 54-56,XX,+X,+4,+6,+9,+10,+14,+15,+17 n=1, 46-47,XY,+4 n=5, 46-47,XY,+4,del(6) n=1, 49,XY,+4,+14,+18 n=1, 50XY,+X,+4,+5,t(9;22),+17 n=1, 50,XY,+X,+4,+5,+17 n=2
Hypodiploidy	10 (11%)	44-45,XY,-7,-10 n=4, 27-29,XY,+4,+6,+10,+8,+12,+18,+21 n=2, 45,XX,-7,t(9;22) n=2, 45,XX,del(6q),-7,t(9;22) n=1, 45,XY,del(6q),-7,t(9;22) n=1

Immunophenotype

B-ALL cases were characterized by the strong positive expression of CD19, CD79a, CD10, CD20, and CD22 B-cell lineage markers. Immaturity markers such as HLA-DR, TdT, and CD34 were expressed in 62 (95%), 65 (88%), and 59 (70%) cases, respectively. Expression patterns of other lineage markers of Ph+ve and Ph-ve cytogenetic groups are given in Table 4.

**Table 4:** Expressions of blast in Ph+ve and Ph-ve cytogenetic group For all CD markers, column 8 indicates the chi-square value calculated using the chi-square test. The percentage in column 3 has been calculated as column 3/column 2. The percentage in column 5 has been calculated as column 5/column 4. The percentage in column 7 has been calculated as column 7/column 6. LAIP, leukemia-associated immunophenotype pattern

Immunophenotype	Overall expression		Ph positive (N = 14)		Ph negative (N = 76)		Test statistics value	p-value
	Total number of cases analyzed	Blast expression, n (%)	Number of Ph+ve cases analyzed	Blast expression in Ph+ve cases, n (%)	Number of Ph-ve cases analyzed	Blast expression in Ph-ve cases, n (%)		
-1	-2	-3	-4	-5	-6	-7	-8	-9
Lineage marker								
CD10	84	80 (95%)	12	11 (91%)	72	69 (96%)	0.3	0.5
CD19	85	85 (100%)	12	12 (100%)	73	73 (100%)	NA	NA
CD34	84	59 (70%)	13	10 (77%)	71	49 (69%)	0.3	0.5
CD38	73	64 (88%)	11	9 (82%)	62	55 (89%)	0.4	0.5
Myeloid antigens								
CD13	76	3 (4%)	12	2 (17%)	64	1 (2%)	6	0.01
CD33	71	20 (28%)	9	3 (33%)	62	17 (27%)	0.13	0.7
CD11b	64	2 (3%)	0	0	53	2 (4%)	0.4	0.5
CD15	54	3 (5%)	0	0	44	3 (8%)	0.7	0.3
LAIP								
CD9	65	58 (89%)	10	10 (100%)	55	48 (87%)	1.4	0.2
CD81	73	69 (94%)	11	10 (91%)	62	59 (95%)	0.3	0.5
CD86	57	32 (56%)	6	5 (83%)	51	27 (53%)	2	0.1
CD73	71	57 (80%)	11	10 (90%)	60	47 (78%)	0.9	0.3
CD44	66	31 (47%)	7	6 (86%)	59	25 (42%)	4.7	0.03
CD58	74	51 (69%)	11	9 (82%)	63	42 (67%)	1	0.3
CD123	62	14 (22%)	9	5 (55%)	53	9 (17%)	6.5	0.01

CD33 was the most frequently observed aberrant marker seen in 20 (28%) cases, followed by CD15 in three (5%), CD13 in three (4%), and CD11b marker seen in two (3%) cases. CD13 was expressed in two (17%) Ph+ve cases and one (2%) Ph-ve cases and was found to be significantly associated with Ph+ve (p = 0.01). The results of other aberrant markers found in Ph+ve and Ph-ve cases are mentioned in Table 4.

Seven leukemia-associated immunophenotype pattern (LAIP) markers, CD9, CD44, CD58, CD73, CD81, CD86, and CD123, were also included in the diagnostic panel. CD9 and CD81 were the most commonly expressed markers in 58 (89%) and 69 (94%) cases. CD73, CD58, CD86, CD44, and CD123 were found in 57 (80%), 51 (69%), 32 (56%), 31 (47%), and 14 (23%) cases, respectively. In our study, for the Ph+ve group, CD123, found in five (55%) (p = 0.01), and CD44, found in six (86%) (p = 0.03) cases, were significantly associated. In Ph-ve cases, the expression of CD123 and CD44 were nine (17%) and 25 (42%), respectively. Other results of LAIP markers are given in Table 4.

Correlation between cytogenetics and immunophenotype

The most crucial correlation found in our study was the aberrant expression of CD33, which was significantly seen in three (33%) Ph+ve cases, followed by CD13 in two (17%) cases (p = 0.01). The values for these markers were 17 (27%) and 1 (2%), respectively, in the Ph-ve group. The percentage expression of CD34 was higher in the Ph+ve group in 10 (77%) cases compared to 49 (69%) cases in the Ph-ve group. The expressions of CD10 and CD38 were lower in 11 (91%) and nine (82%) cases, respectively, in Ph+ve. In contrast, it was higher in 69 (96%) and 55 (89%) cases in Ph-ve, respectively. However, these expressions were not statistically significant. The expression of CD19 was observed at 100% in 12 Ph+ve and 73 Ph-ve cases.

Correlation between cytogenetic groups and clinicopathological parameters

The median age in the Ph+ve group (median age: 30 years) was reported to be significantly higher than the Ph-ve group (median age: 21 years) (p = 0.01). However, no differences were found between the Ph+ve and Ph-ve groups with respect to the proportion of males/females, median hemoglobin level, and percentage of BM blasts. A higher percentage of median WBC count, low platelet count, and low PB blasts were also observed in the Ph+ve group, with no significant differences. Other clinical and hematological parameters are presented in Table 5.

**Table 5:** Baseline clinical and hematological parameters ^a ^Indicates U value calculated using the Mann-Whitney U test ^b^ Indicates the chi-square value calculated using the chi-square test

Variables	All	Ph positive	Ph negative	Test statistics value	p-value
No. of patients, n (%)	90	14 (15%)	76 (84%)	-	-
Age (years), median (range)	23 (13-40)	30 (16-34)	21 (13-40)	315.5^a^	0.01
Male, n (%)	55 (61%)	9 (64%)	45 (60%)	-	-
Female, n (%)	35 (39%)	5 (36%)	31 (40%)	-	-
Male:female ratio	1.57:1	1.8:1	1.48:1	0.07^b^	0.7
Peripheral smear report					
Hemoglobin (g/dl), median (range)	6.5 (2.1-13)	6.5 (2.4-13)	6.5 (1.3-12.8)	505.5^a^	0.8
Leukocyte count (×10^3^/ul), median (range)	10.90 (0.4-365.1)	14.48 (3.81-352.95)	10.76 (0.36-365.1)	430^a^	0.2
Platelet count (×10^3^/ul), median (range)	21.0 (3-321)	19 (3-150)	22 (3-321)	492.5^a^	0.7
Peripheral blast cell %, median (range)	71% (2-97%)	65% (8-96%)	71% (2-96%)	352.5^a^	0.7
Bone marrow blast cell %, median (range)	91% (0.8-98%)	91% (25-97%)	91% (2-98%)	464^a^	0.8
Cytochemistry features					
Sudan Black B (+ve), n	0	0	0	-	-
Periodic Acid-Schiff (+ve), n (%)	17 (19%)	4 (31%)	13 (17%)	-	-
Fibrosis grade					
WHO grade 0, n (%)	1 (1%)	0	1 (1%)	-	-
WHO grade 1, n (%)	3 (3%)	0	3 (4%)	-	-
WHO grade 2, n (%)	1 (1%)	0	1 (1%)	-	-
Lymphadenopathy, n (%)	5 (6%)	3 (23%)	2 (3%)	-	-
Splenomegaly, n (%)	5 (6%)	3 (23%)	2 (3%)	-	-
Hepatomegaly, n (%)	3 (3%)	2 (15%)	1 (1%)	-	-

## Discussion

There are extensive studies on immunophenotypes, karyotypic abnormalities, and their clinical significance for pediatric ALL. However, such studies are very few for AYA and adults [14,18,19]. Prognostication of ALL is based on clinical parameters like age, WBC count, organ dysfunction, and extramedullary involvement. In addition, pathological features like immunophenotype and karyotype are solid prognostic factors [13]. Karyotype independently predicts survival and is associated with other known risk factors in ALL [20].

Ribera et al. reported 21% normal karyotype and 16% t(9;22) (the most frequent structural rearrangement in their study population), followed by hypodiploidy 4% and hyperdiploidy 3% in numerical subgroups [21]. Pullarkat et al. reported 22% normal karyotype and 26% t(9;22) [22]. Moorman et al. found 4-24% t(9;22) and 15% hypodiploidy in 15- to 39-year-old patients [23]. Usvasalo et al. reported 26-29% normal karyotype, 4-8% t(9;22), and 4-18% hyperdiploidy, whereas Moorman et al. reported that the most prevalent specific chromosomal abnormality was t(9;22)(q34;q11) found in 15% of the population. It also reported ploidy subgroups, hyperdiploid and low hypodiploidy, in 7% and 3% of patients, respectively [24,25]. A study conducted by Kadam Amare et al. in the north Indian population in all age groups reported 27% t(9;22) in adults and 6% in children, 24% hyperdiploidy in adults, 44% in children, and 13% hypodiploidy in adults and 8% in children [26]. Another study conducted by Reddy et al. in the southern region of India reported 39.7% normal karyotype, followed by 12.7% hyperploidy and 4.4% t(9;22) [27]. A report from Children’s Oncology Group trial AALL0232 on the largest cohort of AYA to date presented 4.9% t(9;22) in <16 years and 6.3% in >16 years patients [28].

In our study, 51% of cases had a normal karyotype, while t(9;22), seen in 15%, was the most common structural abnormality. Among numerical abnormalities, hyperdiploid was the most typical (17%), followed by hypodiploidy (11%). Thus, the findings of our cytogenetic groups also followed similar trends with earlier published studies where t(9;22) was described as the most prevalent structural abnormality and hyperploidy was the most common numerical abnormality. In our cohort, the incidence of normal karyotype was higher than in earlier published data, possibly due to using a limited FISH panel. The occurrence of Ph positivity in our study was lower than that of earlier reported data, which could be due to a smaller sample size and restricted age group. The incidence of hyperdiploidy shows a decreasing trend from 30-40% in pediatrics (one to 10 years) to <20% in ages between 10 and 15 years, <10% in 15-24 years, and <5% in the 25-44 years age group [29-31]. In our study, only the overall incidence of hyperdiploid (17%) was observed, which is consistent with earlier research. A French ALL Group 93 trial study reported 25% cases of ETV6-RUNX1 in children, 2% cases in adults, and 7% in adolescents [32]. However, in our study, only one case of ETV6-RUNX1 was reported. In previous studies, the prevalence of BCR-ABL1 fusion was associated with higher age [21,22]. A similar result was also found in our study. Data from the Nordic Society of Paediatric Haematology and Oncology 2008 trial reported 1.5-12% cases of iAMP21 in the 1- to 45-year age group [33,34]. However, our study did not find any case with this rearrangement. This could be due to the need for a specific probe/marker to identify this abnormality. Thus, as defined in previous studies, the karyotype is one of the strong prognostic indicators [21]. The implication of finding different cytogenetic abnormalities was helpful in the risk stratification of our study population. This approach enabled better patient management and facilitated the planning of treatment strategies for high-risk patients. The strength of our study is that it revealed the incidence of different chromosomal rearrangements in the AYA study population in this region. In addition, we found an association between Ph+ve karyotype and higher expression of CD13, CD44, CD123, and older age.

Immunophenotype by FCM has been established as one of the most important diagnostic tools, and it has great sensitivity and specificity. It is also less expensive [35]. B-ALL is characterized by the expression of either strong CD19 and another strongly expressed B-lineage marker (i.e., CD79a, CD22, and CD10) or weak CD19 and two other strongly expressed B-lineage markers (14). In studies conducted by Vitale et al. and Velizarova et al. on cytogenetics and immunophenotype, B-ALL was characterized with a similar diagnostic panel of monoclonal antibodies with their expression pattern [36,37]. Our study population’s immunophenotype of B-ALL was consistent with the previous literature, which may mean that there is probably no specific difference in diagnosis of B-ALL immunophenotypic characteristics related to age group as our study was constrained to the AYA population.

Previous studies state that the immunophenotype profile for Ph+ve ALL includes CD25Pos / CD34high / CD10high / CD19high / CD38 weak blasts with dual expression of myeloid antigens CD33 and CD13 [15,35,38]. However, in our study, the expression of CD34 was higher in 10 (77%) cases, CD10 was lower in 11 (91%), and CD38 in nine (82%) cases in the Ph+ve group as compared to the Ph-ve group, which was 49 (69%), 69 (96%), and 55 (89%), respectively. This peculiar surrogate marker profile made a suspected diagnosis of BCR-ABL1 fusion. The implication of these findings was used in deciding the prognosis for those cases where karyotype or molecular study for BCR-ABL1 could not be performed. The level of CD10 expression is a prognostic indicator since it is associated with chromosomal rearrangements [35]. CD10 and CD19 positivity shows good prognosis and characteristics of favorable cytogenetic subgroups such as hyperploidy [39]. The current study showed no significant difference in CD10 expression between Ph+ve and Ph-ve study populations. A study by Thomas et al. reported that the expression of CD34 has been recognized as a favorable prognostic factor in children. However, in adults, it was identified as an adverse prognostic factor [40]. In our study, the Ph+ve group known for adverse prognosis was associated with a higher percentage of CD34.

In previous studies, the overall expression of MyAg markers in B-ALL was reported to be 4.3% to 64% [41]. Velizarova et al. reported CD13 as 60%, Bhushan et al. reported CD33 as 39%, and Gupta et al. reported CD13 as 25.6% [14,37,42]. According to Seegmiller et al., the most frequently expressed antigen was CD13 (54.5% of cases), followed by CD33 (43.0%), CD15 (36.0%), and CD11b (20.0%) [15]. In the current study, MyAg expression of CD33 and CD15 were the most common aberrantly expressed antigens in 20 (28%) and three (5%) cases, respectively. CD13 was found in three (4%) cases, and CD11b was expressed in two (3%) cases. MyAg CD33 and CD13 were commonly expressed in three (33%) and two (17%) cases of the Ph+ve cytogenetic group, respectively. However, in Ph-ve patients, CD33 and CD13 were expressed as 17 (27%) and 1 (2%) cases, respectively, which is in line with the earlier data [35,43]. The presence of immunophenotypic aberrancies also confirms the existence of different genetic entities in our cohort. The most common MyAg in previous studies was CD13, followed by CD33. In contrast, CD33 was most common in our setting, followed by CD13, which could be due to a restricted age population, a smaller sample size, and different fluorochromes.

As reported in previous studies, the association between MyAg expression and high-risk cytogenetic group Ph+ve leads to poor prognosis [14,15,37,42]. However, a study by Vitale et al. observed no correlation between aberrant expression and the Ph+ve group and their clinical outcomes [36]. Patients with CD33 overexpression tend to have an inferior prognosis. However, CD33 expression was not found to be an independent predictor of survival [35]. CD33 expression was also more frequent at relapse than at initial diagnosis, which may reveal clonal evolution [44]. Our study also observed a correlation between the Ph+ve group and MyAg expression. As discussed, MyAg’s role is to identify negative prognoses, and this association can be validated by monitoring patients’ clinical outcomes in the future.

LAIP markers CD44 and CD123 also showed significant differences between the Ph+ve and Ph-ve groups. The expression of LAIP markers CD123 and CD25 is emerging as unique biological markers that can identify the disease, its prognosis, and targeted therapy [45]. However, in the current study, CD25 was not included in the panel. During treatment, drug-induced changes in immunophenotype may lead to false negative results in MRD studies. To overcome this problem, it was recommended that multiple aberrant markers found to represent LAIP at diagnosis be followed so that at least one LAIP would remain stable [46]. Thus, adding these markers in MRD panels can increase the assay’s sensitivity by distinguishing leukemic lymphoblasts from their regular counterparts. Coustan-Smith et al. reported a marginal association (p = 0.09) between CD86 expression and hyperdiploidy [47]. A higher expression level of CD86 was detected in three BCR-ABL1-positive cases reported by Sędek et al. [48]. Tembhare et al. reported that in MRD-positive samples, CD73 had a maximum (83%) frequency of LAIP, and CD86 had the highest (100%) stability of aberrant expression, whereas CD58, CD123, and CD44 were found to be equally relevant in the detection of frequency of LAIP in MRD samples. Also, CD44 was significantly associated with a Ph+ve group [49]. The overexpression of several markers in hyperdiploid (51-65 chromosomes) ALL cases, including CD86, CD97, and CD123, was confirmed by Djokic et al. [50]. Jain et al. reported that CD73 was aberrantly expressed in 90.41% of cases and CD86 in 60.87% of B ALL cases [51]. However, in our study, in five (83%) cases, the expression of CD86 was seen in Ph+ve and in 27 (53%) cases in the Ph-ve group. CD123 was observed in five (55%) cases of Ph+ve (one case was hyperdiploidy) and nine (17%) cases in Ph-ve patients.

As per earlier studies, along with karyotype and immunophenotype, older age and higher WBC counts were also associated with the Ph+ve group to determine the prognostic impact [22,31]. In our settings, the Ph+ve group was significantly associated with older median age (p = 0.012), CD13 (p = 0.01), CD44 (p = 0.03), and CD123 (p = 0.01). Other baseline parameters did not show any difference between the groups.

The limitation of this study is that some markers were unavailable during the processing of a particular case. While expressing the results of different markers in percentage, such cases have been excluded in the denominator where these markers were not performed. For example, as seen in Table 4, from a total of 90 cases, CD13 and CD33 were performed respectively in 76 and 71 cases only (column 2). Positive expression was found in three and 20 cases for CD13 and CD33, respectively. If the marker had been performed for more cases, more comprehensive data showing an association between cytogenetic and immunophenotype could have been reported.

## Conclusions

In this study, we found the incidence of different cytogenetic abnormalities in the AYA population in South India and also observed the association of MyAg, LAIP, and baseline parameters with Ph+ve cases. The uniqueness of this study is that it was centered on AYA, which is recognized as a critical population. The data found in this study also provides valuable information and emphasizes the need to do a more elaborate study for this age group in this region. As the current study was for a limited number of cases and adopted a limited FISH and CD marker panel, the further comprehensive study should include a higher number of patients, a larger marker panel, and antibodies to further reinforce the correlation between other baseline parameters, immunophenotype, and cytogenetics for AYA to improve the survival rate for this age group.

## Appendices

Standard operating procedure

Day 1: Culture Setup

1. Bone marrow samples were collected in a sodium heparin vacutainer, labeled with the patient’s name and hospital ID.

2. Upon receipt, the WBC count was checked, and the sample volume was calculated as 7.5 divided by cell count in millions​.

3. The media was prewarmed, and 5 ml of RPMI, 3 ml of FBS, and 100 µl of antibiotics were added to culture vials (T25 flasks) labeled ONC and ON.

4. The calculated sample volume was added to both ONC and ON vials, with 100 µl of colcemid added only to the ONC vial.

5. The culture setup was performed in a laminar airflow hood under strict sterile conditions.

6. The culture vials were transferred to a CO_2_ incubator (37°C with 5% CO_2_) and incubated for 24 hours.

7. Culture vials were loosely capped.

8. The following morning, 100 µl of colcemid was added to the ON vial and incubated for 30 minutes.

Day 2: Harvesting

1. At the designated harvesting time, the vials were removed from the incubator.

2. Added 100 µl of colcemid to the ON vial, returned it to the incubator, and set the timer for 1 hour.

3. During this hour, prepared a hypotonic solution (KCl) and kept it prewarmed at 37°C. Prepared the fixative and kept it at room temperature.

4. After 1 hour, the vials were removed from the incubator, transferred to 15 ml centrifuge tubes, and spun for 10 minutes at 1000 RPM.

5. After spinning, the supernatant was aspirated using a pipette vacuum pump. The pellet was tapped, and KCl was added drop by drop (initially 1 ml) while tapping or gently vortexing. Then, KCl was added more quickly, totaling 10 ml per tube.

6. The tubes were placed in a water bath at 37°C for 10 minutes. Ensure the KCl column was fully immersed.

7. After 10 minutes, the tubes were removed from the water bath. Added ten drops of prepared fixative to each tube and gently tilted to mix. The tubes were then spun in a centrifuge for 10 minutes at 1000 RPM.

8. After centrifugation, the supernatant was discarded without disturbing the pellet. The pellet was tapped, and fixative was added drop by drop (initially 1 ml) while tapping gently or using a vortex mixer. After the first 1-2 ml, fixative was added more quickly.

9. After adding the fixative, the tubes were refrigerated at 4°C for 24 hours before slide preparation.

Day 3: Slide Preparation

1. Removed the tube from the fridge and allowed it to reach room temperature (at least 20 minutes).

2. Spun the tube for 10 minutes. Added 5 ml of fixative to the tube and spun again for 10 minutes.

3. Checked the pellet for clarity and whiteness.

4. Washed glass slides were dipped in a Coplin jar containing methanol-1 and methanol-2, then swirled in chilled Milli-Q water.

5. The slide was held horizontally, and two drops of cell suspension were gently added at a 20-30° angle.

6. Added four to six drops of fixative across the slide to overlap with the cell suspension drops.

7. Drained the slide by blowing air onto it, wiped the back side, and placed it on a slide warmer at 37°C for 30 seconds.

8. Prepared three to four slides per case and labeled them with the patient’s lab number.

9. Examined the slides under a phase contrast microscope.

10. Placed the slides in an oven at 90°C for 20 minutes to age them. After aging, proceeded with G-banding.

Day 4: Banding

1. After aging, removed the slides for banding.

2. Prepared 50 mg of trypsin using 50 ml of PBS and warmed it in a water bath.

3. Stored the PBS in the refrigerator to halt trypsin activity.

4. Immersed the slide in the prewarmed trypsin solution for 10 seconds.

5. Transferred the slide into cold PBS after the trypsin treatment.

6. Stained the slide with working Giemsa stain and examined it under the microscope for banding patterns.
